# Quantitative Analysis of Four Catechins from Green Tea Extract in Human Plasma Using Ultra-Performance Liquid Chromatography-Tandem Mass Spectrometry for Pharmacokinetic Studies

**DOI:** 10.3390/molecules23040984

**Published:** 2018-04-23

**Authors:** Jeong-Eun Park, Tae-Eun Kim, Kwang-Hee Shin

**Affiliations:** 1 Research Institute of Pharmaceutical Sciences, College of Pharmacy, Kyungpook National University, Daegu 41566, Korea; knu_park@knu.ac.kr; 2 Department of Clinical Pharmacology, Konkuk University Medical Center, Seoul 05029, Korea; tekim@kuh.ac.kr

**Keywords:** green tea, epigallocatechin-3-gallate (EGCG), epicatechin-3-gallate (ECG), epigallocatechin (EGC), epicatechin (EC), ultra-performance liquid chromatography-tandem mass spectrometry (UPLC-MS/MS)

## Abstract

Green tea is consumed as a beverage worldwide and has beneficial effects, such as a lower risk of cardiovascular disease and cancer. A quantitative analysis of the beneficial components in plasma is important for understanding the potential health benefits of green tea. Four catechins—epigallocatechin-3-gallate (EGCG), epicatechin-3-gallate (ECG), epigallocatechin (EGC), and epicatechin (EC)—which account for the majority of the components of green tea, were analyzed by ultra-performance liquid chromatography-tandem mass spectrometry (UPLC-MS/MS). In this study, a validated method was optimized to obtain the blood concentrations after the one-time ingestion of 630 mg green tea extract with digoxin and then after the ingestion of 630 mg green tea repeatedly for 15 days. The calibration curve, including the LLOQ, was constructed over 1–500 ng/mL for EGCG, ECG, and EGC and 0.1–50 ng/mL for EC. The method for inter- and intra-validation was applied, acceptable for both accuracy and precision. We successfully developed an appropriate UPLC-MS/MS method for human plasma with good reproducibility and sensitivity. Thus, this method could be applied for future preclinical and clinical studies on EGCG, ECG, EGC, and EC.

## 1. Introduction

Green tea is a widely consumed beverage worldwide and for a few decades now, the scientific benefits of drinking green tea have been well known [1,2]. The positive effects of green tea on the human body include a reduction in the risk of cardiovascular disease, cancer prevention, and antihypertensive and antioxidative activity [3,4,5]. Catechins, which are polyphenol compounds that account for the majority of tea components, are known to constitute approximately 30–42% of brewed green tea ingredients [6,7]. Daily One green tea extracts (Atlantic Essential Products, Hauppauge, NY, USA), which are easily obtainable health supplements, contain approximately 70% catechins. Among the green tea catechins, the four major polyphenols are epigallocatechin-3-gallate (EGCG), epicatechin-3-gallate (ECG), epigallocatechin (EGC), and epicatechin (EC) [8]; EGCG is the most abundant catechin and constitutes approximately 50–80% of the total catechin content [6,9,10]. Owing to the popularity of green tea, it is necessary to understand the health effects of the constituents of green tea.

Furthermore, the interactions of catechins with drugs should be considered if green tea is consumed while taking medication. P-glycoprotein (P-gp) is a transport enzyme that is responsible for the efflux of a variety of drugs and directly affects the efficacy of natural products or chemotherapeutic drugs [11]. In particular, digoxin is known to be a representative substrate drug for P-gp [12], and pharmacokinetic changes are expected when administered with an inhibitor or inducer of P-gp. Previous studies have suggested that EGCG and EGC [13,14] were potential P-gp substrates and that EC [11] enhanced P-gp function. Quantification of the major catechins of green tea extract in the human plasma is important to understand both the beneficial effects of green tea and the drug interaction effect derived P-gp transporter. We conducted a quantitative analysis of four catechins in human plasma from a clinical study to investigate the interaction of green tea with digoxin, a representative substrate of P-gp.

In the quantitative determination of catechins, liquid chromatography-mass spectrometry has been used for previous studies, but the analysis method requires a long chromatographic analysis time of approximately 40 min [15]. Although other studies have demonstrated the quantification of green tea components in a 15-min analysis using liquid chromatography-tandem mass spectrometry, the quantitative curves used were set to the same concentration ranges and did not consider that each major component may be present in different amounts in the body [15,16]. In another study, different concentration curves were adjusted for each catechin, unfortunately there has been no analysis of the simultaneous quantitation of the most abundant catechins, such as EGCG [17].

The present study has reported a valid quantification method to determine four catechins (EGCG, ECG, EGC, and EC) in human plasma by using sensitive ultraperformance liquid chromatography (UPLC) coupled with tandem quadrupole mass spectrometry. Using this method, we determined the four plasma catechin concentrations after a single dose of green tea extract and repeated administration of green tea extract in the presence of digoxin, which is a known P-gp substrate, in healthy male subjects.

## 2. Results and Discussion

### 2.1. Optimization of the UPLC-MS/MS Conditions

**Figure 1:**
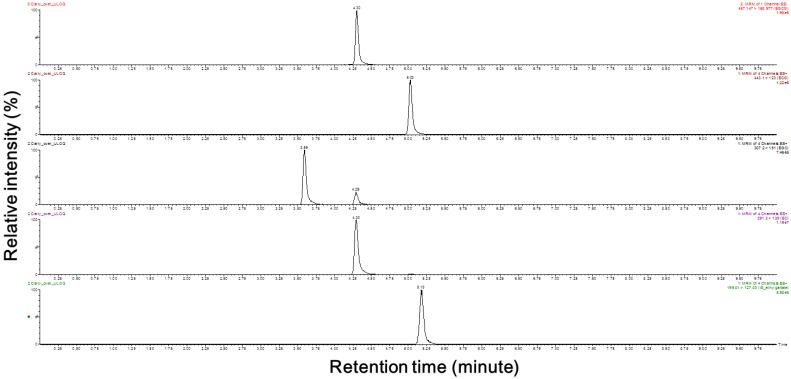
Chromatograms of epigallocatechin-3-gallate, epicatechin-3-gallate, epigallocatechin, epicatechin and ethyl gallate. EGCG, epigallocatechin-3-gallate; ECG, epicatechin-3-gallate; EGC, epigallocatechin; EC, epicatechin. The selected chromatographs were representative of the highest quantitation limit.

The purpose of the present study was to develop a method for the simultaneous quantification of four catechins in human plasma. The total analysis run time was 10 min in this experiment, which represents a 4-fold reduction compared to that reported in another study [8]. As described in the Materials and Methods section, an Atlantis T3 column (4.6 mm × 50 mm, 3 µm) from Waters Acquity^TM^ was used to separate the mixed compounds. The retention times for the elution of EGCG, ECG, EGC, EC, and the ethyl gallate (internal standard, I.S.) were 4.30, 5.03, 3.59, 4.30 and 5.18 min, respectively. Overall, a good resolution was achieved for ECG, EGC, and the I.S., but the difference in elution time between EGCG and EC was only 0.01 min, and the peak was detected at a similar time. However, through the sensitive detection capability provided by using precursor ions and product ions, we detected four catechins and ethyl gallate (the I.S.) in human plasma (Figure 1). The detector was operated in multiple reaction monitoring (MRM) mode using the transitions of *m*/*z* 457.15 → 168.98 for EGCG, 443.10 → 123.00 for ECG, 307.20 → 151.00 for EGC, 291.20 → 139.00 for EC, and 199.01 → 127.03 for ethyl gallate.

To maximize the ionization response, various mobile phase conditions were tested. In the reverse-phase, line (A) was immobilized with water, and the organic solvent of line (B) had better peak shape when acetonitrile was used instead of methanol. When polyphenol compounds are analyzed on a reverse-phase column, it is common to adjust the pH to below 3 by the addition of acetic acid or formic acid [18]. The peak shapes for the four catechins in acetic acid as the mobile phase were better than those obtained when formic acid was used as the mobile phase. And a lower pH was expected to reduce tailing, but the peak was best detected at pH 3. When polyphenol compounds are analyzed, separation using a gradient eluent is commonly used [18], and the mobile phase gradient method was established by reference to a previous study [19].

Using the optimized UPLC-MS/MS conditions, our preliminary analysis aimed to establish a human plasma preparation method. One of the simplest methods of plasma pretreatment is protein precipitation (PP) using organic solvents [20]. Methanol and acetonitrile are the most commonly used organic solvents for this purpose owing to their low cost and the small number of steps required to develop the sample preparation method [21]. First, the protein removal ability was evaluated when two organic solvents were added at 10 times the volume of the plasma. Visual inspection revealed that the protein was more condensed and precipitated after the addition of acetonitrile rather than methanol; however, the signal-to-noise ratio was very low with both solvents, and the shape of the peak was not properly detected. These results, which could be interpreted as peripheral interference peaks, were eliminated by the addition of an organic solvent but also by dilution of the target analytes; eventually, the sensitivity of the analytes decreased. Second, to increase the sensitivity, organic solvent was added, and 80% of the supernatant was dried and redissolved to concentrate the supernatant, followed by UPLC-MS/MS. We expected a higher sensitivity than the first method; however, the signal-to-noise ratio was below 5, and the peak was not detected. As there was no extraction step for the target analytes, the sensitivity was expected to be low. Therefore, the extraction method of the four catechins from plasma was optimized from that described in an existing study, with slight modifications [19]. The plasma liquid-liquid extraction (LLE) pretreatment method with ethyl acetate used in the study was described in Section 3.

### 2.2. LLOQ, Selectivity and Calibration Curve Linearity

**Figure 2:**
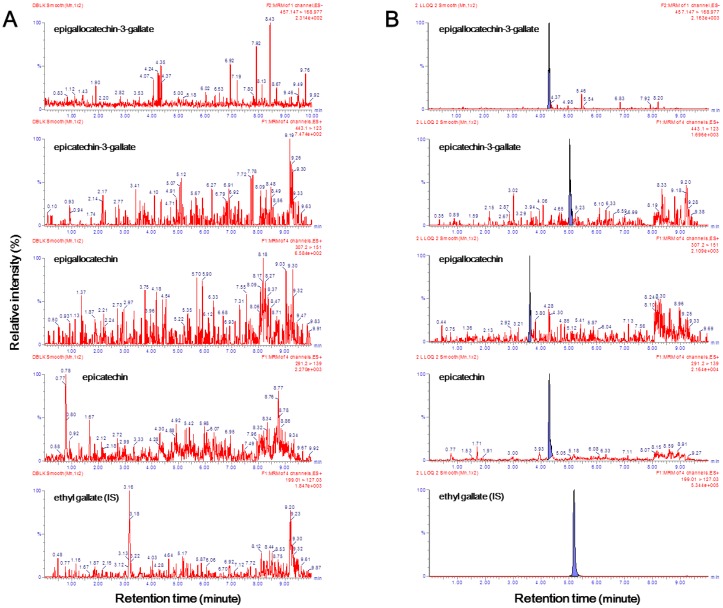
Representative chromatograms of (**A**) double blank human plasma and (**B**) spiked with EGCG, EGC, EGC, and EC at the LLOQ. EGCG, epigallocatechin-3-gallate; ECG, epicatechin-3-gallate; EGC, epigallocatechin; EC, epicatechin. Signal-to-noise ratios for EGCG, ECG, EGC, and EC were 76.93, 20.69, 14.16 and 54.70, respectively in Figure 2B.

Chromatograms of samples of double blank plasma (free of both analytes and I.S.) and lower limit of quantification (LLOQ) analytes and the I.S. in blank plasma are illustrated in Figure 2. It has been proven that endogenous components that interfered in the double blank plasma samples did not affect the retention time of EGCG, ECG, EGC, EC, and ethyl gallate (Figure 2A). As there was no interference of endogenous substances, even in the six independent blank plasma samples, the selectivity criteria could be fulfilled. The signal-to-noise ratios exceeded 10 for all four catechins (Figure 2B). The LLE method was chosen to maximize the ion response. As the LLOQ of the four compounds had a higher signal-to-noise ratio than the set in this study, the extraction method was judged to be appropriate. In particular, in this experimental method, the LLOQ of EGCG, EGC, and ECG was 1 ng/mL, and the LLOQ of EC was 0.1 ng/mL. Similar to a previous study, different concentration curves were used because the concentration of EC was remarkably low in human plasma [15]. The results of this analysis indicated that the sensitivity of the method used in this study was 10 times higher for EGCG, EGC, and ECG or 100 times higher for EC than that of a previously reported UPLC with single quadruple mass spectrometry method [22]. Additionally, the LLOQ of EGCG, EGC, ECG, and EC were 3.8 times, 8.7 times, 7.8 times, and 39 times lower than that obtained in the previously reported LC with triple quadrupole mass spectrometry method [16].

The method showed good linearity, including at the LLOQ values (1 ng/mL for EGCG, ECG, and EGC and 0.1 ng/mL for EC). The typical linear equations with 1/*x* weighting were *y* = 0.21242*x* − 0.259584 (R^2^ = 0.9994) for EGCG, *y* = 0.181501*x* − 0.0416934 (R^2^ = 0.9994) for ECG, *y* = 0.125654*x* − 0.0445765 (R^2^ = 0.9960) for EGC, and *y* = 1.94133*x* − 0.109767 (R^2^ = 0.9989) for EC. Using the LLE method that was repeated, which was a double extraction process, the sensitivity and curve linearity were obtained in the concentration ranges 1–500 ng/mL for EGCG, ECG, and EGC and 0.1–50 ng/mL for EC.

### 2.3. Accuracy and Precision

The accuracy is the agreement between the actual values and the measured values and precision is the agreement between the independent experimental results [23]. The accuracy and precision of inter- and intra-day measurements for the four catechin quality control samples evaluated in plasma are summarized in Table 1.

All accuracies and precisions of the four analyte concentrations, repeated five times on each of the other 3 days, were within acceptable limits. Specifically, the accuracies of the inter-day and intra-day measurements were in the ranges of 90.38–108.2% and 88.85–111.3%, respectively. The precisions calculated by the RSD of inter- and intra-day measurements were in the ranges of 4.52–9.77% and 1.45–10.6%, respectively. The accuracy and relative standard deviation (RSD) percentage were within the appropriate threshold range (three quality control samples of ±15% and LLOQ of ±20%), which proved that this method was repeatable and reproducible.

### 2.4. Stability

The acceptance criterion for stability was ±15% [24,25] difference compared with the reference samples that were prepared by the conventional pretreatment method, without any artificial treatment (Table 2). 

When repeated three times consecutively at the low and high concentrations established in this study to evaluate the stability under various test conditions, the reinjection stability, stability after 24 h in autosampler conditions, and processed sample stability (PSS) were estimated, and all values fell within the threshold of ±15%. These results confirmed that stability was inside the autosampler, even if the sample was analyzed overnight. The quality control (QC) plasma prepared at low and high concentrations was completely frozen (−80 °C) and melted three times and then compared with freshly prepared spiked QC samples. Samples subjected to one and two freeze-thaw cycles were also analyzed to evaluate the differences between the samples that received three freeze-thaw cycles (labeled as test) and reference samples. All four catechins were found in the acceptable range after the freeze-thaw cycles. Therefore, it was indicated that the plasma samples stored at −80 °C were stable after three cycles of the freeze-thaw process. The short-term and long-term stabilities were evaluated in the spiked plasma and working stock solutions. The short-term stability was confirmed by the analysis of blood plasma and stocks after 6 h at room temperature, equivalent to the time required to analyze the samples. The long-term stability was obtained after storage for 52 days at 4 °C for the stocks and −80 °C for the plasma samples. The results confirmed that the stock and plasma samples were stable at room temperature for 6 h. The plasma samples were stable at −80 °C for 52 days; however, the stocks were not stable at 4 °C. Although the stocks of EGCG and ECG were stable at 4 °C for 52 days, the high-concentration QC samples of EGC and EC exceeded the acceptable deviation from the reference samples (15.80% and 20.50%, respectively). When the analytes were dissolved in 50% methanol solution and stored in the refrigerator, the concentration of the solution after 52 days was higher than that of the reference because the solvent did not freeze and instead evaporated.

### 2.5. Recovery, Matrix Effect and Hemolysis

The mean recoveries of epigallocatechin-3-gallate, epicatechin-3-gallate, epigallocatechin, and epicatechin are presented in Table 3. In this study, because the plasma was pretreated using the LLE method, a better extraction efficiency than simple PP was expected. The LLE method was designed using ethyl acetate as the extraction solvent with a better satisfactory extraction ability than diethyl ether [26,27]. The extraction efficiencies of EGCG, ECG, EGC, and EC at the low, middle, and high QC concentrations were 75.4 ± 1.55, 65.5 ± 3.66, 64.3 ± 1.84 and 61.2 ± 1.55, respectively.

The matrix effect was analyzed to determine whether the composition of the biological sample affected the response of the analytes or internal standards. The variation coefficient was within 15% when evaluated with respect to the peak areas of analytes and the internal standards from six different biological samples prepared in low and high concentrations. The coefficient of variation (CV) among the six different blank plasma samples that were spiked at low and high concentrations was negligible for the assay, which proved that there was no effect of the biological sample on the analytes or internal standard substances.

The hemolysis test was evaluated to determine if red blood cells that had not been removed in the course of processing whole blood into plasma affected the pharmacokinetic assay. In vitro, hemolysis can occur as a result of careless handling during the separation processes and centrifugation steps [28]. When the iron-containing protein, hemoglobin, is released into the plasma or other intracellular components escape into the surrounding fluid, false quantitation or dilution of the analytes may occur [29]. To investigate the effect of hemolyzed samples on the analysis of pharmacokinetic parameters, blank plasma with artificially mixed whole blood was prepared with a low concentration of QC. The mean difference between the triplicate analysis of hemolysis samples and reference samples was within the critical range (±15%) between −1.32 and 2.93. Therefore, it was concluded that the presence of hemoglobin and other substances from the red blood cells did not affect the analysis results.

### 2.6. Dilution Integrity

The dilution integrity is used to prove that if the concentration of the sample exceeds the range of the calibration curve, dilution does not affect the analysis (Table 4). The stocks were prepared at a concentration equivalent to five times the upper limit of quantification (ULOQ) and diluted 1/5 and 1/50 times with the same solvent. 

After a 1/5 dilution, the accuracy of the four catechins was found to range from 91.04% to 105.8%; for a 1/50 dilution, the accuracy of analytes was found to range from 91.55% to 110.7%. The precision of all the QC samples was in the range of 2.05–10.1% (1/5 concentration) and 0.11–3.57% (1/50 concentration). The accuracy and precision of the two concentrations of the four catechins were 85–115% and ≤15% of the nominal concentrations, respectively. Therefore, the extrapolation was valid for the sample concentrations that exceeded the calibration curve up to fifty-fold.

### 2.7. Pharmacokinetics

**Figure 3:**
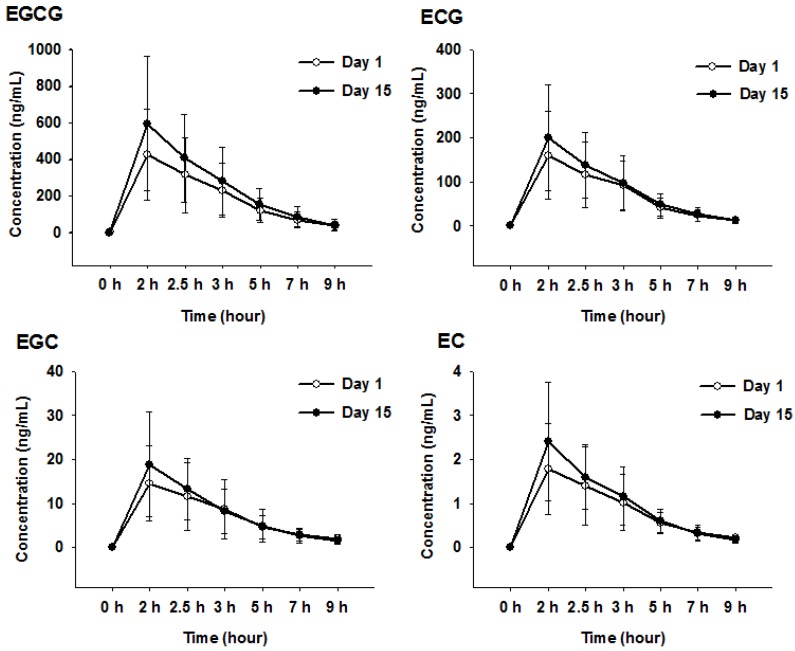
Mean plasma concentration-time profiles of four catechins after oral administration of 630 mg of green tea extract to healthy volunteers given once and continuously administered for 15 days. EGCG, epigallocatechin-3-gallate; ECG, epicatechin-3-gallate; EGC, epigallocatechin; EC, epicatechin. Day 1: Orally administered 630 mg of green tea extract with 0.5 mg digoxin in sixteen volunteers; Day 15: Repeated daily administration for 15 days of 630 mg green tea extract with a single administration of digoxin in fifteen volunteers.

The mean changes of the four catechins in human plasma are plotted in Figure 3, and the corresponding pharmacokinetic parameters are summarized in Table 5. All four catechins in human plasma had the highest concentrations at approximately 2 h after oral administration. The results demonstrated that all four catechins showed a tendency for increased area under the concentration curve from the zero time-point to the last measurement (AUC_0–9_), the AUC from the zero time-point to infinity (AUC_0–∞_), and the maximum observed concentration (C_max_) after repeated administration for 15 days, but the difference was not statistically significant. The results of the present study suggested that repeated administration of green tea could affect the pharmacokinetic changes associated with elimination of EGC and EC in catechins. Interestingly, after repeated dosing of 630 mg green tea extract, the elimination half-life (T_1/2_) of EGC and EC was decreased to 0.74- and 0.70-fold the values obtained on day 1, respectively. 

A recent study suggested that P-gp could involve the absorption and elimination of green tea [30], and in the current study, repeated dosing of the catechins appeared to reduce the T_1/2_ of ECG and EC. Large inter-individual variabilities for PK parameters were observed, and the degree of exposure to the body varied greatly with each catechin, which would be expected due to the differences in absorption and drug transport between individuals.

## 3. Materials and Methods

### 3.1. Reagents and Materials

EGCG, ECG, EGC, and EC were purchased from Toronto Research Chemicals (Toronto, ON, Canada). Ascorbic acid, ethylenediaminetetraacetic acid (EDTA), sodium phosphate monobasic, ethyl acetate, and ethyl gallate (internal standard, I.S.) were obtained from Sigma-Aldrich (St. Louis, MO, USA). Acetonitrile and methanol (Merck, Darmstadt, Germany) were HPLC grade. Acetic acid was purchased from Duksan (Gyeonggi-do, Korea).

### 3.2. UPLC-MS/MS Instruments and Conditions

The UPLC-MS/MS system was composed of an Acquity^TM^ UPLC system (Waters, Milford, MA, USA) equipped with a column compartment, refrigerated autosampler, binary solvent manager, and tandem quadrupole mass spectrometer with an API source (XEVO TQ-MS) connected to MassLynx software version 4.1 (Waters, Milford, MA, USA). The separations were conducted on an Atlantis T3 column (4.6 mm × 50 mm, 3 µm) from Waters maintained at 40 °C. The mobile phase was composed of 0.3% acetic acid in water (A) and 0.3% acetic acid in acetonitrile (B), and the following gradient elution was applied: 2% B at 0 min, 2–30% B at 0–4 min, held at 30% B for 3 min, 30–98% B at 7–7.1 min, 98% B until 8 min, 98–2% B at 8–8.1 min, and 2% B until 10 min. The process was conducted with an injection volume of 5 µL and a flow rate of 0.5 mL/min. The following parameters were adjusted to determine the maximized ionization: mass spectrometry desolvation temperature and gas flow were set at 625 °C and 1100 L/h, respectively; the selective ion monitoring mode was [M + H]^+^ ions for all catechins and ethyl gallate, except for EGCG, which used [M − H]^–^ ions. The detected ion cone voltages and collision energy in each electrospray mode was 25 V, 25 V for EGCG; 25 V, 15 V for ECG; 20 V, 15 V for EGC; 20 V, 15 V for EC; and 18 V, 12 V for ethyl gallate, respectively.

### 3.3. Preparation of the Calibration Curve and Sample Pretreatment

The stock solutions of EGCG, ECG, EGC, and EC were prepared at 1 mg/mL in 0.2% ascorbic acid and 0.005% EDTA in distilled water. The stock solutions were diluted using 50% methanol to concentrations of 10, 50, 100, 250, 500, 2500, and 5000 ng/mL, except for EC, which was diluted to 1, 5, 10, 25, 50, 250, and 500 ng/mL. The LLOQ of the four materials was set to be equal to the lowest concentration of the calibration curve. LLOQ (10 ng/mL) and low (200 ng/mL), medium (400 ng/mL), and high (1000 ng/mL) quality control samples were diluted by the same method (for EC, the quality control samples were 1, 20, 40, and 100 ng/mL, respectively). Ethyl gallate was dissolved in 50% methanol to a concentration of 1 mg/mL and then serially diluted to a final concentration of 1 µg/mL in the same solution. All the above solutions were stored at 4 °C.

Before the analyte was spiked, the blank plasma and ascorbic acid-EDTA solution (0.1% EDTA and 20% ascorbic acid in 0.4 M NaH_2_PO_4_ buffer) were mixed at a ratio of 10:1, and then, the mixed blank plasma aliquot was stored at −80 °C until analysis. The stock solution (10 µL) was spiked into mixed blank plasma (90 µL), and 10 µL ethyl gallate solution (1 µg/mL) was added. Ethyl acetate (1.0 mL) was added to extract the four catechins from the plasma. The samples were vortex-mixed for 15 min and then centrifuged at 1900× *g* for 20 min at 4 °C. The supernatant was transferred into another polypropylene tube, and the same procedure was repeated for the remaining precipitate. The organic layer was condensed to dryness by using a centrifugal vacuum concentrator (Labconco Corp., Kansas City, MO, USA) for 60 min at 35 °C. After solvent evaporation, the residue was reconstituted in 100 µL of 0.3% acetic acid in 15% acetonitrile. The reconstituted solution was vortex-mixed for 15 min and centrifuged at 16,200× *g* for 10 min at 4 °C. The supernatant was transferred to a vial, and a 5 µL aliquot of the residual solution was injected into the UPLC-MS/MS system for analysis.

The EGCG, ECG, and EGC final calibration curve concentrations were 1 (equal to LLOQ), 5, 10, 25, 50, 250 and 500 ng/mL, and the low, medium and high QC sample concentrations were 20, 40 and 100 ng/mL, respectively. The EC plasma calibration curves were prepared from concentrations of 0.1 (equal to LLOQ), 0.5, 1, 2.5, 5, 25, and 50 ng/mL, and the concentrations of the low, medium and high QC samples were 2, 4, and 10 ng/mL, respectively. The concentrations in human plasma of the abovementioned four analytes were determined as the peak area ratio of the internal standard.

### 3.4. Method Validation

The selectivity was evaluated by the analysis of blank human plasma (i.e., no catechins and I.S.) from six different sources. The interfering substances present in the biological sample were detected at the peak retention time of the analytes based on the LLOQ. At the LLOQ, relative to the blank, the area of the peaks detected during the retention time satisfied the criteria of 20% or less for analytes and 5% or less for internal standards.

The plasma calibration curves and five replicated LLOQs and the low, medium, and high QC samples were analyzed on three different days. The linearity of the three calibration curves measured on each different day satisfied both R ≥ 0.9950 and R^2^ ≥ 0.9801 and was generated from the analyte peak area ratio relative to the I.S. using a weighted linear regression (1/*x*). The LLOQ of the analyte spiked into blank plasma was assessed by a signal-to-noise ratio of 5:1. The inter- and intra-day precision was expressed as the RSD (%) and the accuracy was expressed as the percentage of the measured concentration value of the nominal concentration value. A precision ≤15% and accuracy within ±15% were deemed to be acceptable, except for the LLOQ, which required a precision ≤20% and an accuracy ±20%.

The matrix effects on the four catechins and I.S. were assessed at two concentrations (low and high) using plasma with six different origins. The peak area ratio of target analytes relative to the internal standard was selected based on the RSD values of ≤15% at both low and high concentrations. The extraction recovery of the analytes in human plasma was repeated three times at the low, medium, and high concentrations, and the RSD value of the peak area ratio at each concentration was less than or equal to 15%. The recovery was calculated from the division of the peak area ratio after the average of three extractions at each concentration by the ratio of the peak area before extraction and reported as a percentage.

Carryover was evaluated through the injection of the highest concentration of the calibration curve and the subsequent measurement of blank plasma samples. This experiment was conducted to confirm whether analytes and I.S. substances were present in blank samples after the analysis of high concentration samples. The carryover was considered not to affect the analysis when the detection area of the analyte interference peak was within 20% of the LLOQ and within 5% of the I.S. peak area.

The stability of the four catechins in human biological samples was established using blank plasma spiked with low and high QC concentrations, in triplicate. The stability was studied under five different conditions as follows: (1) samples stored in an autosampler at 4 °C for 24 h and repeatedly injected (re-inject stability); (2) new samples maintained at 4 °C for 24 h in the autosampler conditions and then injected (processed sample stability); (3) samples subjected to three cycles of −80 °C for more than 12 h and thawed at 18–21 °C (room temperature) (freeze and thaw stability); (4) stock solutions (prior analyte spiking) maintained at room temperature for 6 h, at 4 °C for 52 days, and compared with freshly prepared samples (stock and working solution stability, short-term and long-term, respectively); and (5) plasma (after analyte spiking) maintained at room temperature for 6 h, at −80 °C for 52 days, and compared with freshly prepared samples (sample stability, short-term and long-term, respectively). The long-term stability evaluation of 52 days was based on the 50 days from the date of the first blood sampling to the end of the analysis. All the stability evaluations were repeated three times, and the acceptable deviation criteria were set to within ±15% of the samples prepared on the given day for each concentration.

The dilution integrity was assessed through the dilution of the samples five times and 50 times with a sample that was five times above the ULOQ (500 ng/mL for EGCG, ECG, and EGC; 50 ng/mL for EC). Five replicate experiments were conducted at each concentration. The dilution integrity criteria were deemed acceptable when the precision was ≤15% and the accuracy was within ±15%.

A total of 20 μL of whole blood was added to 980 μL of blank plasma to prepare hemoglobin to remain in the plasma, and the analysis of the low concentration sample was repeated three times. When comparing the concentration of the test sample containing whole blood with that of the reference sample prepared in accordance with the conventional method, hemolysis was judged to be unaffected in the plasma analysis if the variation was within 15%.

The method of validation was performed according to the Food and Drug Administration guidelines for Bioanalytical Method Validation and Korea Ministry of Food and Drug Safety guidelines for bioanalytical method validation [24,25].

### 3.5. Pharmacokinetic Analysis

The quantitative method described above was used to investigate the pharmacokinetics of four catechins in the human plasma after oral administration of a single dose of 630 mg green tea extract (Daily One, Atlantic Essential Products) and 0.5 mg of digoxin under the fasting state. After repeated oral dosing of 630 mg green tea extract for 15 days, 0.5 mg of digoxin was administrated orally once on the Day 15 and the plasma concentration was analyzed. The 630 mg Daily One green tea extract used in the study contained 300 mg of catechin. Sixteen healthy adult volunteers were administered 630 mg green tea extract 1 h before the ingestion of 0.5 mg digoxin; this was termed day 1. After 24 h, the 630 mg of green tea extract was repeatedly administered daily for 15 days to fifteen volunteers. All participants were supplied with an explanation of the purpose and content of the study and provided voluntary written consent to participation in the study. The protocol of this study was approved at the Institutional Review Boards (IRBs) and all of the human biological samples were collected at Konkuk University Medical Center, Seoul, Korea (IRB No. KUH1280094). For the calculation of pharmacokinetic parameters, blood samples were collected before dosing (fasting blood samples) and at 2, 2.5, 3, 5, 7, and 9 h after the administration of the green tea extract. Day 1 biofluid samples were obtained after a single dose of 0.5 mg digoxin and 630 mg green tea extract. Day 15 samples were collected after the ingestion of 0.5 mg digoxin and 15 days of repeated dosing of green tea extract. Given the high frequency of use of green tea [1,2], it is expected that there will be drug interactions with digoxin, which is a representative substrate of p-gp responsible for the transport of several drugs [12]. For this reason, the representative substances in green tea were quantified after the combination treatment with digoxin, a known P-pg substrate. The plasma samples of all volunteers were stored in a blood collection container containing a 10:1 ratio of EDTA-K_3_ anticoagulant and ascorbic acid-EDTA solution and stored below −70 °C until analysis. The noncompartmental pharmacokinetic parameters were calculated. The C_max_ and the time to Cmax (T_max_) were obtained from the observed data. The AUC_0–t_ and AUC_0–∞_ were calculated using the linear/log trapezoidal method. The T_1/2_ was calculated as ln (2)/λz. λz was the elimination rate constant slope of the plasma concentration-time plot calculated using linear regression.

### 3.6. Statistical Analysis

The analysis of the statistical significance of the pharmacokinetic parameters analyzed at two treatment periods was computed using SPSS (IBM SPSS Statistics 22, Chicago, IL, USA). The T_max_, C_max_, AUC_0–t_, AUC_0–∞_, and T_1/2_ of the four catechins were compared on day 1 (single-dose green tea with digoxin) and day 15 (repeated-dose green tea with digoxin). The pharmacokinetic parameter data were evaluated as follows: non-normal distribution when the *p*-value was less than 0.05 after the Kolmogorov-Smirnov test and normal distribution when the *p*-value was 0.05 or more. The Wilcoxon signed rank test and Student’s *t*-test were used for the analysis of non-normal distribution and normal distribution data, respectively.

## 4. Conclusions

In the study, a UPLC-MS/MS method was developed for the determination of four green tea catechins (EGCG, ECG, EGC, and EC) in heathy human plasma after a single oral administration of 630 mg green tea extract and after repeated administration for 15 days. The double extraction method was sensitive, with 1 ng/mL LLOQ for EGCG, ECG, and EGC and 0.1 ng/mL LLOQ for EC when using the repeated liquid-liquid extraction. The validation data were successfully implemented with sufficient repeatability and reproducibility. The current method would be recommended applied for the pharmacokinetic evaluation of catechins or related drug-drug interaction studies in human.

## Figures and Tables

**Table 1:** Inter-day accuracy, intra-day accuracy, and precision of epigallocatechin-3-gallate, epicatechin-3-gallate, epigallocatechin, and epicatechin in human plasma.

Analytes	Nominal Concentration (ng/mL)	Inter-Day (*n* = 5)			Intra-Day (*n* = 5)		
		Calculated Concentration (ng/mL)	Accuracy (%)	Precision (%)	Calculated Concentration (ng/mL)	Accuracy (%)	Precision (%)
EGCG	1 (LLOQ)	1.01 ± 0.0922	101	9.16	0.976 ± 0.103	97.7	10.6
	20 (Low QC)	18.5 ± 0.845	92.4	4.52	18.4 ± 0.682	92.1	3.71
	40 (Medium QC)	39.3 ± 3.84	98.3	9.77	37.5 ± 1.03	93.7	2.74
	100 (High QC)	91.2 ± 4.22	91.2	4.63	90.1 ± 2.74	90.1	3.04
ECG	1 (LLOQ)	1.03 ± 0.0879	104	8.54	1.05 ± 0.0994	109	9.43
	20 (Low QC)	18.9 ± 1.18	94.4	6.27	19.3 ± 0.281	96.6	1.45
	40 (Medium QC)	38.3 ± 2.26	95.8	5.90	40.4 ± 1.41	101	3.49
	100 (High QC)	90.8 ± 3.43	90.4	3.78	90.1 ± 1.64	88.9	1.82
EGC	1 (LLOQ)	1.08 ± 0.0817	108	7.55	1.11 ± 0.0722	111	6.50
	20 (Low QC)	19.9 ± 1.45	99.7	7.31	19.9 ± 1.02	99.3	5.16
	40 (Medium QC)	38.4 ± 2.51	96.1	6.52	38.8 ± 3.59	97.0	9.25
	100 (High QC)	96.4 ± 5.65	96.4	5.86	96.5 ± 5.23	96.6	5.42
EC	0.1 (LLOQ)	0.103 ± 0.00976	101	9.44	0.110 ± 0.0071	102	6.43
	2 (Low QC)	1.86 ± 1.13	93.2	7.04	1.86 ± 1.13	93.1	6.79
	4 (Medium QC)	3.74 ± 0.243	93.5	6.52	3.75 ± 0.366	93.7	9.77
	10 (High QC)	9.49 ± 0.436	94.9	4.60	0.976 ± 0.103	97.7	4.91

EGCG, epigallocatechin-3-gallate; ECG, epicatechin-3-gallate; EGC, epigallocatechin; EC, epicatechin; The measured concentration values are shown as the means ± standard deviation; precision (%) calculated as a percentage of the standard deviation divided by the average.

**Table 2:** The percentage of change difference in stability of epigallocatechin-3-gallate, epicatechin-3-gallate, epigallocatechin, and epicatechin under various conditions at two concentrations (*n* = 3).

	EGCG		ECG		EGC		EC	
Concentration (ng/mL)	20.0	100	20.0	100	20.0	100	2.00	10.0
Stability	% change							
Reinjection	11.4	8.91	8.82	1.99	0.31	9.57	−0.36	7.64
Autosampler for 24 h	7.36	6.88	-4.84	6.02	−4.23	7.81	12.2	14.7
One freeze-thaw cycle	−1.02	−2.45	−2.01	2.29	−5.44	−8.90	5.28	1.76
Two freeze-thaw cycles	3.99	−1.02	1.49	8.27	3.46	7.46	12.9	13.7
Three freeze-thaw cycles	2.08	0.015	0.496	0.55	−5.95	−2.00	−0.18	−2.88
Plasma at room temperature for 6 h	−8.87	8.12	1.84	−9.38	−4.31	5.33	10.0	0.41
Stock at room temperature for 6 h	−10.6	13.2	−2.12	13.3	−3.61	−5.41	8.26	13.5
Plasma at −80 °C for 52 days	9.96	4.61	4.25	−2.51	13.94	12.7	7.36	1.16
Stock at 4 °C for 52 days	−1.82	−0.52	0.72	9.99	−11.6	15.8	9.39	20.5

EGCG, epigallocatechin-3-gallate; ECG, epicatechin-3-gallate; EGC, epigallocatechin; EC, epicatechin; % change, percent difference compared to newly prepared samples at each concentration. All stability assessments were compared newly prepared at low and high concentrations of QC to the following conditions. Reinjection, samples stored in an autosampler at 4 °C for 24 h and repeatedly injected; autosampler for 24 h, new samples maintained at 4 °C for 24 h in the autosampler conditions and then injected; freeze-thaw cycle, samples subjected to three cycles of −80 °C for more than 12 h and thawed at 18-21 °C; plasma at room temperature for 6 h, plasma (after analyte spiking) maintained at room temperature for 6 h; stock at room temperature for 6 h, stock solutions (prior analyte spiking) maintained at room temperature for 6 h; plasma at −80 °C for 52 days, plasma (after analyte spiking) maintained at 4 °C for 52 days; stock at 4 °C for 52 days, stock solutions (prior analyte spiking) maintained at −80 °C for 52 days.

**Table 3:** Summary of the recovery, matrix effect, and hemolysis for the assay method (recovery and hemolysis, *n* = 3; matrix effect, *n* = 6).

	EGCG			ECG			EGC			EC		
Concentration (ng/mL)	20.0	40.0	100	20.0	40.0	100	20.0	40.0	100	2.00	4.00	10.0
Recovery from spiked plasma (%)	74.9	74.1	77.1	64.1	62.6	69.6	64.2	62.5	66.2	61.4	59.6	62.7
Matrix effect RSD (%)	12.3	–	3.64	5.79	–	4.61	8.60	–	8.18	7.28	–	6.36
Hemolysis change (%)	2.93	–	–	−2.02	–	–	−1.32	–	–	−1.99	–	–

EGCG, epigallocatechin-3-gallate; ECG, epicatechin-3-gallate; EGC, epigallocatechin; EC, epicatechin; RSD, relative standard deviation calculated as a percentage of the standard deviation divided by the average; Hemolysis change (%), percentage difference compared with newly prepared samples using blank plasma at low quality control concentration.

**Table 4:** Dilution integrity at a concentration five-fold higher than the upper limit of the quantification concentration.

	EGCG		ECG		EGC		EC	
Above ULOQ (ng/mL)	2500						250	
Dilution concentration (ng/mL)	50.0	500	50.0	500	50.0	500	5.00	50.0
Mean ± SD (ng/mL)	45.8 ± 1.64	506 ± 51.0	49.9 ± 0.0545	529 ± 25.0	49.3 ± 0.91	456 ± 9.35	5.53 ± 0.11	45.7 ± 2.43
Accuracy (%)	91.6	101	99.7	106	98.5	91.0	111	91.5
RSD (%)	3.57	10.1	0.11	4.72	1.85	2.05	1.97	5.32

EGCG, epigallocatechin-3-gallate; ECG, epicatechin-3-gallate; EGC, epigallocatechin; EC, epicatechin; ULOQ, upper limit of quantification; The measured concentration values are shown as the means ± standard deviation; RSD, relative standard deviation calculated as a percentage of the standard deviation divided by the average.

**Table 5:** Pharmacokinetic parameters of four catechins after oral administration of 630 mg green tea extract.

Analytes	Analysis Type	AUC_0–9_ (h ng/mL)	AUC_0–∞_ (h ng/mL)	T_max_ (h)	C_max_ (ng/mL)	T_1/2_ (h)
EGCG	Day 1	1356 ± 779	1487 ± 841	2.00	424 ± 250	2.33 ± 0.712
	Day 15	1791 ± 1008	1960 ± 1053	2.00	596 ± 368	2.45 ± 1.46
	*p*	^a^ 0.192	^a^ 0.181	-	^a^ 0.143	^b^ 0.820
ECG	Day 1	502 ± 291	539 ± 307	2.00	160 ± 99.7	2.12 ± 0.467
	Day 15	589 ± 317	630 ± 327	2.03 ± 0.129	201 ± 120	2.17 ± 0.767
	*p*	^a^ 0.435	^a^ 0.435	^-^	^a^ 0.308	^a^ 0.800
EGC	Day 1	51.2 ± 29.1	64.5 ± 30.5	2.00	14.6 ± 8.55	4.51 ± 2.81
	Day 15	56.3 ± 30.3	65.2 ± 30.5	2.00	18.9 ± 11.8	3.35 ± 2.47
	*p*	^b^ 0.211	^b^ 0.532	-	^b^ 0.112	^b^ 0.041
EC	Day 1	6.15 ± 3.11	7.20 ± 3.16	2.00	1.79 ± 1.04	3.32 ± 1.80
	Day 15	7.13 ± 3.25	7.77 ± 3.35	2.03 ± 0.129	2.44 ± 1.30	2.32 ± 0.931
	*p*	^a^ 0.398	^a^ 0.626	-	^b^ 0.053	^b^ 0.031

Each of the four catechin pharmacokinetic parameters is expressed as the mean ± standard deviation. EGCG, epigallocatechin-3-gallate; ECG, epicatechin-3-gallate; EGC, epigallocatechin; EC, epicatechin; AUC_0–9_, area under the concentration curve from the zero time-point to the last measurement; AUC_0–∞_, area under the concentration curve from the zero time-point to infinity; T_max_, time of maximum concentration; C_max_, maximum observed concentration; T_1/2_, half-life. Day 1, oral administered of 630 mg green tea extract with 0.5 mg digoxin in sixteen volunteers; Day 15, repeated daily administration for 15 days of 630 mg green tea extract and a single administration of digoxin in fifteen volunteers; ^a^ *t*-test; ^b^ Wilcoxon signed-rank test for calculated statistical significance.
